# On Being Open in Closed Places: Vulnerability and Violence in Inpatient Psychiatric Settings

**DOI:** 10.1111/nup.70005

**Published:** 2024-11-14

**Authors:** Cat Papastavrou Brooks, Isobel Johnston, Erinn Gilson

**Affiliations:** ^1^ Population Health Sciences, Bristol Medical School The University of Bristol Bristol UK; ^2^ SPIRED Clinic Sussex Partnership NHS Foundation Trust Brighton UK; ^3^ School of Social Sciences The University of Manchester Manchester UK; ^4^ Department of Humanities Merrimack College North Andover Massachusetts USA

**Keywords:** inpatient, mental health, power, psychiatry, resistance, violence, vulnerability

## Abstract

High levels of violence and conflict occur in inpatient psychiatric settings, causing a range of psychological and physical harms to both patients and staff. Drawing on critiques of vulnerability from the philosophical literature, this paper contends that staff's understanding of their relationship with patients (including how they should respond to violence and conflict) rests on the dominant, reductive account of vulnerability. This account frames vulnerability as an increased susceptibility to harm and so regards ‘invulnerable’ staff's responsibility to be protecting and managing vulnerable patients. We offer an alternative view of vulnerability as an openness and capability to be changed, which illuminates how the common account of vulnerability is used to justify staff's coercive power over patients and to control staff behaviour. Our main argument is that staff's adoption of this negative approach to vulnerability is associated with a range of factors that are connected to the violence and conflict endemic to these settings. Staff's need to situate themselves as invulnerable and therefore incapable of harm, we argue, leads to significant issues through: damaging staff ability to emotionally regulate; coercing patients into an asymmetrical openness leading to aggression to restore status; damaging therapeutic relationships by enforcing separation between staff and patients; increasing staff's reliance on unhelpful and rigid techniques (such as de‐escalation); repressing staffs’ ability to learn and grow through encounters with patients. Finally, we offer recommendations for how vulnerability and openness could be cultivated as a relational and radical practice in spaces that are traditionally closed and hostile to it.

## 1 Introduction

Inpatient psychiatric settings are characterised by high levels of violence (Hallett and Dickens 2021). This includes patient violence towards staff, staff abuse of patients, self‐harm and suicide, and restrictive practices such as seclusion and restraint (Bowers et al. 2009; Busch, 2000). Violence in inpatient psychiatric settings puts both staff and patients at risk of harms such as injury, psychological trauma, staff burnout and sickness, and patient deaths during restraint (Allen et al. 2019; Larue et al. 2013; Muir‐Cochrane, O'Kane, and Oster 2018).

The common account of the normative relationship between inpatient psychiatric staff and the patients (often involuntarily detained) in their care is that staff are responsible for protecting and caring for patients, whose vulnerability is understood as an increased susceptibility to harm (Sellman 2005). However, to preserve their own sense of invulnerability, staff emotionally distance themselves from others labelled as vulnerable and attempt to mask or suppress their own vulnerability. This avoidance of vulnerability, we argue, increases violence in these settings, is damaging to therapeutic relationships between psychiatric inpatient staff and patients, and poses a substantial barrier to patient recovery. Crucially, we argue that the recommended intervention for reducing violence in psychiatric settings—training staff in de‐escalation (NICE., 2005)—is counterproductive insofar as it reinforces this understanding of the staff/patient relationship and exacerbates avoidance of vulnerability.

The central claim of this paper is, therefore, that the accepted account of vulnerability as susceptibility to harm is used to justify and uphold the power that inpatient psychiatric staff exercise over patients, contributing significantly to the violence and resulting harms ubiquitous in these settings. Perhaps counterintuitively, interacting with vulnerable patients from a stance that regards vulnerability as merely susceptibility to harm is counterproductive and exacerbates the harms that patients (and staff) experience.

## 2 Conceptualising Vulnerability

Within the philosophy of nursing literature there is a broad consensus that the practice of nursing exists as a response to the heightened vulnerability of patients, who are more susceptible to harm due to a higher exposure to risk or a reduced capacity to protect themselves (Sellman 2005). Debates centre around:

1. If an individual is vulnerable regardless of whether they are aware of their vulnerability (Clarke and Driever 1983, p. 2010)
2. If vulnerability is caused by intrinsic characteristics or structural forces excluding mentally ill people from society (Paradis‐Gagné and Pariseau‐Legault 2020)
3. Whether our understanding of susceptibility to harm should be broadened to include damage to patients’ social and epistemic capacities (Kidd and Carel 2017)
4. The extent to which vulnerability interferes with human flourishing (Carel 2009; Sellman 2005)
5. The causes of any vulnerability nurses experience (Sellman 2005)

However, it is clear from a summary of these points of debate that they share an underlying assumption that vulnerability is a characteristic primarily possessed by patients and, because of its connection with susceptibility to harm, it holds a negative value.

This common view of vulnerability also ties it to an array of other conditions and qualities: weakness, dependency, powerlessness, incapacity, deficiency, passivity, incompetence, and, especially pertinent, lack of control (Gilson 2013). Seeing vulnerability in this way enables people to limit its significance by framing it as an exceptional and negative state separate from the normal course of human life and leading to undesirable dependence on others or to otherwise impaired capacities. This devaluation of vulnerability results in attempts to avoid it by pursuing an impossible ideal of an individualistic and impervious self and by repositioning vulnerability as primarily a characteristic of those occupying lower social positions, for our purposes, psychiatric patients. In this context, vulnerability is most strongly associated with incompetence, a lack of control, and dependency. Given these specific connotations, devaluing those who are vulnerable as lesser and vulnerability as ‘to‐be‐avoided’ can lead to regarding vulnerable others as threatening (Gilson 2021, p. 20; Lorey 2015).

In contrast with this reductive negative conception, it is more accurate and fruitful to recognise the ambiguity of vulnerability and the diverse ways it is experienced. As a general and shared condition, vulnerability may be understood in terms of plasticity and openness and, in that sense, be defined as being open to being affected and affecting in ways that cannot necessarily be anticipated or controlled (Gilson 2013). This includes the possibility of being affected through being harmed, however harm is just one potential amongst a multiplicity of other transformations brought about, which can also include learning, empathy and connecting with others. Thus, vulnerability is inherently relational: what makes a person vulnerable in specific ways are the kinds of relations they have.

This aligns with a common view across therapeutic modalities of the self as relational. Accordingly, therapeutic change occurs in the context of a relationship, that between mental health professional and patient. Further, therapeutic change happens as the patient develops a new relationship with themselves, others, their ‘history’ and their emotions. A relational perspective shifts our understanding of the self from 'the independent, ideally autonomous, and rational agent […] into an interdependent, vulnerable, and emotional agent' (Metz and Miller 2016, 7). This notion of the self is most obvious in evidence‐based therapies derived from psychoanalytic approaches such as Cognitive Analytic Therapy, where shifting the roles one takes in different interactions is foundational for recovery (Ryle and Low 1993), and mindfulness therapies that emphasise self‐compassion (Gilbert 2009). However, even in cognitive approaches therapeutic alliance is a strong mediator of change (Baier, Kline, and Feeny 2020), and the importance of finding more adaptive ways to relate to others and your own emotional life is centred.

A relational view of the self also aligns with a relational approach to ethics, which underlies the analysis in this paper. There are various features that make an ethic a relational one, as Metz and Miller point out. Two such features are helpful for this analysis. First, a relational ethic considers relationships among people as one of the most important 'units of moral concern' (Metz and Miller 2016, 7). Second, when assessing why actions are right or wrong, a relational ethic focuses on the ways people relate to one another: are they caring, attentive, and empathetic, and is the relationship 'characterised by sympathetic responsiveness' or are they insensitive, dominating, and thus 'relationship‐withering' (Metz and Miller 2016, 7)? In the clinical context, '…relational ethics refers to occasions when a determinative factor in the morality of an action is the nature and quality of the relationship between the clinician and the patient' (Olsen 2021, 57).

Thus, there are clear reciprocal connections among notions of self that are found in the therapeutic literature, an account of vulnerability as openness rather than simply as susceptibility to harm, and a relational approach to ethics. By reframing vulnerability as a condition of openness, it is possible to take stock of and distinguish different experiences of, and ways of relating to, vulnerability (Gilson 2021), including the kinds of vulnerability that lead to injury or therapeutic progress. This latter form of vulnerability is fundamentally a practice and, as such, must be cultivated and developed within a relationship (even if that relationship is with yourself). On the other hand, without opening yourself up to be altered and affected, it is impossible to form the kinds of relationships with others, self, and emotions that make therapeutic progress possible.

It is helpful here to distinguish between physical and emotional vulnerability. Much of the popular literature is concerned with emotional vulnerability, which—despite negative perceptions of vulnerability in general—is more likely to be positively viewed as connected to innovation, creativity, change, and closeness with others (Brown, 2012). By contrast, physical vulnerability is more often linked to illness, disease, being assailable, and risk of death (Boldt 2019). Because of its importance to relationships, emotional vulnerability is the focus in much of this paper, alongside physical and epistemic vulnerability. It is unsurprising, given human embodiment, that both physical and emotional vulnerability are closely linked in inpatient psychiatric settings. We will argue that, despite the analytical distinction between them, their connection means it is not possible for staff to cultivate one without the other in their relationships with psychiatric patients.

## 3 ‘Othering’ the Vulnerable in Mental Health Settings

Theoretical accounts within the philosophy of nursing reflect an understanding of vulnerability as the boundary between staff and patients that is common to clinical practice within mental health services. When vulnerability is seen as a characteristic of patients (and one that increases risks of harm) then invulnerability becomes a professional ideal continually sought by staff. The phenomenon can be understood on an individual level; because patients are seen to occupy a lower social position, and staff are keen to distance themselves from traits associated with them, vulnerability is avoided. However, it can also be understood structurally; through framing mental health patients’ vulnerability in terms of a lack of capacity, weakness, susceptibility to harm, and being in need of protection, staff as a group are able to justify the coercive power they hold over patients, particularly in inpatient settings, where patients are often detained and treated against their will (Chan 2002; Keown et al. 2018). If staff are also perceived as vulnerable, and thus patient‐like, the coercive power of the psychiatric system is called into question, undermining not only an individual staff member's place within the system, but the system itself.

Given that vulnerability is a key feature of human life, ensuring that clinical staff in mental health settings consistently present as invulnerable requires an ideological system to coercively regulate their behaviour. This operates through either punishing any displays of staff vulnerability as unprofessional (and therefore threatening their continued employment) or by attempting to convince them that any emotional openness will lead to them or their colleagues being exploited or harmed by patients. Emotional vulnerability is forestalled by connecting it to the prospect of threatening forms of physical vulnerability.

### 3.1 Staff Vulnerability as Unprofessionalism

Although there is a general consensus that staff disclosing their own mental illness can be helpful in forming therapeutic relationships with patients and reducing stigma (Marino, Child, and Campbell Krasinski 2016; Victor et al. 2022), substantial barriers exist to their doing so. A key factor negatively affecting mental health professionals’ willingness to share their lived experience in the workplace is how disclosures to colleagues may result in being seen as impaired professionals, resulting in pressure to conform to the idea of an 'invulnerable professional' to not have their fitness to practice questioned (Johnston et al. 2022; King et al. 2020; Oates, Drey, and Jones 2017). Other research found that the idea of professional boundaries was often weaponized against staff with lived experience of mental health issues (i.e. any disclosure necessarily involved a boundary violation which was burdensome to the patient) despite the fact that disclosures, when managed appropriately, tend to be viewed positively by both parties (Audet 2011; Baca 2011; Gutheil and Gabbard 1998).

Staff's anxieties about being perceived as unprofessional if they display emotion is a valid one insofar as it reflects the predominant values of a culture—internal to inpatient settings and that of culture in general—that devalues vulnerability. So, it is vital to acknowledge the ambiguity of vulnerability: for staff, being vulnerable with patients is both valuable, conducive to good care, and potentially harmful (insofar as it opens staff to charges of unprofessionalism and the possible repercussions thereof, perhaps including poor evaluations of their work and potentially losing their jobs or even professional registration). It could also go against the expectations of patients, who might also see clinical roles necessitating that clinicians present themselves as invulnerable.

### 3.2 Staff Vulnerability as a Reversal of Power

This phenomenon is best documented in forensic settings, where there is substantial anxiety that being open or vulnerable in any way with patients (e.g., sharing minor details of your life) opens staff to manipulation and exploitation by patients (Bowers et al. 2009). This view results in recommendations that clinicians should understand their own vulnerabilities to prevent patients from exploiting them (Richmond et al. 2012), that that self‐disclosure increases risks of harm not only to staff but also 'the people close to them' (Rachwal and Gredecki 2024) and that even empathising with patients is dangerous because it might lead to sharing personal information which could put them at risk (Goodman et al. 2020). These recommendations highlight how staff are trained to conceptualise their own emotional and physical vulnerability as connected. The contention is that sharing parts of yourself and your life with patients and letting your guard down, through being emotionally vulnerable, brings the risk of disclosing information that might put either yourself or your colleagues at risk (e.g., them knowing what neighbourhood you live in leading to the risk of stalking, or being convinced to smuggle something onto the ward for a patient i.e., later utilised as a weapon). Therefore, under this ideological framework, the only way of assuring physical safety is through maintaining emotional distance.

On the surface, it might seem impossible for patient vulnerability to mean both that they are susceptible to harm (as a result of being weak, passive, dependent and injurable) whilst also being able to cause harm (as a result of being active, strong and injuring). However, this perception ignores the specific and nuanced way in which ideas of vulnerability are applied to psychiatric patients, occurring within a multi‐dimensional conceptual space as opposed to a continuum between strong and weak. The key here is the way in which patient vulnerability is related to dependency, incompetence or incapacity, and lack of control. When regarded as especially vulnerable and susceptible to harm, patients are also perceived as out‐of‐control and liable to cause harm, which leads staff to believe that they must maintain control for everyone's safety (Johnston et al. 2022) and thus maintain their own invulnerability so as not to add to the lack of control and likelihood of harm. So, the reversal of power (in staffs’ perceptions) is also a transformation of the dynamic from one that is under control (because staff are in control and acting authoritatively) to one that is out of control (because patients, who are not capable, are manipulating staff and staff are not in control of them). Given this way of perceiving patient vulnerability, staff are anxious to maintain power.

### 3.3 Social Factors

Further context can be provided by considering the impact of social position on who is either able to be vulnerable or perceived as vulnerable by others in psychiatric inpatient settings. There are significant disparities between racial groups in the likelihood of being subject to seclusion and restraint (Bond, DiCandia, and MacKinnon 1988; Frueh et al. 2005; Schnitzer et al. 2020). Evidence suggests that this is partly because the behaviour of black patients is more likely to be seen as dangerous and requiring restrictive practices (Spector 2001) due to racial bias, with staff finding it more difficult to engage empathically with groups that are feared within society (Brown, 2012; Baldessarini et al. 2009; Warner 2007). Brown argues that there is a juxtaposition of danger/threat and vulnerability in the way mental health patients are portrayed, particularly when those individuals come from racialized or socially marginalised groups (Brown, 2012; Warner 2007). The assumption that vulnerable groups of people are more likely to be dangerous aligns with the associations vulnerability has in this setting with incapacity and being out‐of‐control.

Systemic oppression also plays a key role in which staff face the most pressure to disavow or mask their own vulnerability. Narrow definitions of clinical professionalism often contain racial biases and are used to disadvantage staff who are members of racially minoritized groups (Dave et al. 2021, p. 2). These groups also face the additional burdens of negotiating clinical professionalism in often white‐dominated workplaces (Powers et al. 2016; Schafir 2022). The increased precarity of professionalism afforded to staff from racially minoritized groups could make it even more difficult for them to engage in the practice of vulnerability without facing more serious repercussions than their white counterparts. For different structural reasons, women – particularly younger or less experienced women—might also have additional pressures placed on them to pursue invulnerability in this environment. Female staff on psychiatric wards are seen not only as more vulnerable to physical violence from patients (despite the reverse being true (Lawoko, Soares, and Nolan 2004; Renwick et al. 2019) but also more susceptible to being 'un‐boundaried' or too emotionally open with patients (Goodman et al. 2020), in ways connected to stereotypes of women as being weaker, more emotional, and passive. In very different ways, being a vulnerable professional might pose additional problems for racially minoritized groups and women; for the former because their professionalism is constantly being undermined from multiple sides, creating an additional cost to expressing vulnerability, and for the latter because they are already seen as more vulnerable, meaning that any expressions of vulnerability are compounded.

## 4 Invulnerability and Violence

Seeing vulnerability as an openness to being changed (as opposed to just a susceptibility to being harmed) illuminates difficulties with an apparently diverse group of practices currently existing in inpatient psychiatric environments. These result from a renunciation or avoidance of, an anxiety concerning, or a hostility towards vulnerability within staff teams and significantly contribute to conflict and violence in these settings.

### 4.1 The Pursuit of Invulnerability and Emotional Regulation

The perceived conflict between professionalism and being seen as emotionally vulnerable in psychiatric environments results in a demand for staff suppression or masking of emotions in the workplace (Hochschild 2012, pp. 37–42). Emotional suppression is a form of emotional avoidance, where one's own emotionally expressive behaviour is consciously inhibited or masked (Gross and Levenson 1993), leading to changes in how those emotions are experienced. This is in contrast to a deeper level of emotional processing, which involves confronting, working through, and finding ways of dealing with difficult emotions. Although emotional suppression can often be adaptive in enabling individuals to deal with stressful situations, long term it has negative consequences for how individuals manage to regulate their emotional states, including being able to experience a range of positive emotions (and not become stuck in cycles of negative emotion), to change their emotional states in response to new situations or demands, as well as not to feel ‘overwhelmed’ by difficult emotions (Iwamitsu et al. 2005). Given the high incidence of violence and staff trauma in psychiatric environments, these attempts to avoid being labelled as unprofessional might require masking of complex and difficult emotions for prolonged periods in the presence of both patients and other staff. Emotional suppression has a significant impact on emotional regulation abilities. A systematic review found that repeated emotional suppression was related to negative physiological outcomes and less experience of positive affect, as opposed to deeper processing strategies such as emotional reappraisal (Gross 2002, p. 20). Other research has shown that being in environments where vulnerability is perceived as a weakness constrains individual abilities to emotionally regulate (Borelli et al. 2019). This could explain the well‐documented connection between psychiatric staff's emotional suppression and their increased risk of burnout (Edward, Hercelinskyj, and Giandinoto 2017). In addition to this cost to staff, emotional suppression has been found to result in a reduction in interpersonal responsiveness as well as negative views of patients and hostile behaviour (Butler, Lee, and Gross 2007). Yet, the importance of emotional regulation has been particularly emphasised in staff responses to aggression on psychiatric wards, with staff members who were able to regulate their emotions effectively and respond 'to the situation and not their own frustration' being seen as more effective in de‐escalating conflict (Roberton et al. 2012).

### 4.2 Coerced and Asymmetrical Openness

Whilst they are undergoing inpatient psychiatric treatment, patients are often required to be open with healthcare professionals about a range of issues including mental illness, trauma and their current emotional state. Perceived lack of engagement with professionals can result in the curtailment of leave and lengthier inpatient stays against patient wishes (Harris, Lechevallier, and Buhagiar 2021).

This demand is exacerbated in forensic settings where patients are often required by the court to engage in psychological evaluation and treatment, which then contributes to the criminal justice process (Perlin 1991; Simms‐Sawyers, Miles, and Harvey 2020). Disclosures to an individual staff member (with whom the patient might have a positive relationship) are disseminated to the wider team through notes and reports without consent. This practice can be understood as a form of coerced openness and vulnerability; patients have no control over the ways in which their emotions and histories are open to the perceptions of others. This paternalistic practice stems from regarding patients in their vulnerability as incompetent dependents and is oriented toward acquiring increased control, not only to protect patients themselves but also (particularly in forensic settings) to protect the public. This required and/or coerced vulnerability is in stark relief to staff's lack of emotional openness or disclosure in these settings; a member of staff will often know incredibly personal details of a patient's history before meeting them, whereas even after lengthy interactions staff are often unwilling to be open about even small features of their own lives. Unsurprisingly, this asymmetry and lack of choice on the part of patients has significant consequences for the development of a trusting therapeutic relationship between staff and patients.

There is an established link between the shame, humiliation, and coercion arising from admission to psychiatric hospital and patient violence as a means of restoring status (Gilligan 2003). There are multiple aspects of this experience that may elicit shame in patients, however, it is plausible that the asymmetry and coercion present in disclosures between staff and patients is a potential driver of patient violence as a way of equalising this imbalance. Notably, the attempts discussed in the second section of this paper around staff beliefs that they should remain closed off as a way of preventing patients from manipulating or even harming them could conversely increase the risk of patient violence. It is worth noting that these are other specific experiences of vulnerability, albeit harmful ones, so we might note that these additional harmful experiences emerge in a context in which vulnerability is treated as undesirable and vulnerable people are treated coercively, due to this reductive understanding of vulnerability. Aversion to emotional vulnerability on the part of staff potentially precipitates an increase in the emotional vulnerability of patients and, consequently, the physical vulnerability of all to violence.

### 4.3 Preserving Separation From Patients

Staff unwillingness to be vulnerable to patients is not limited to disclosures of mental health issues. Staff can be generally unwilling to engage in any form of interaction with patients aside from those explicitly required by their role as nursing staff. Although the importance of authentic and reciprocal interactions between staff and patients is overwhelmingly emphasised in the literature on de‐escalation (Berring, Pedersen, and Buus 2016; Goodman et al. 2020; Johnston et al. 2022), many staff do not engage in shared and mutual activities with patients (Shattell, Andes, and Thomas 2008; Thibeault et al. 2010). Such activities enable patients to gain a sense of staff members as people in an otherwise often formal and cold relationship, where 'staff member's humanness comes with vulnerability; an exposing of the self that fosters the relationship' (Hartley, Redmond, and Berry 2022).

Although time‐constraints play a significant role in this phenomenon, there is also a fear on the part of staff of seeming too close or too similar to patients, and a refusal to engage in more reciprocal or normal interactions with patients is a way of managing this fear (Peternelj‐Taylor 2004). However, this strategy also prevents the formation of mutual and authentic relationships that can reduce incidents of violence and make it easier to manage conflict when it occurs (Bowers et al. 2009; Espinosa et al. 2015).

This is also a key instance where physical and emotional vulnerability are intertwined. It might be argued that, given the risks of physical violence ubiquitous on psychiatric wards, that although staff should aim for increased emotional openness with patients, they should attempt to do so in a way that does not compromise their physical safety.

It is worth saying first, that, aside from remaining in the ward office and not interacting with patients, inpatient psychiatric staff (and indeed most healthcare staff), are limited in how much they can guarantee physical safety; anything which reduces the risk of being physically harmed also necessarily involves an increased physical distance from patients. This is problematic for two reasons: firstly, it is practically difficult to build a relationship with someone if you do not spend time physically close to them. Secondly‐ and more interestingly—anything which communicates to patients that you do not trust them enough to be physically vulnerable with them is a de facto barrier to the reciprocal emotional openness necessary for relationship building. This idea is backed up by the empirical literature; staff inadvertently communicating their fears for their physical safety around patients can increase the risk of patients being violent towards them (Johnston et al. 2022), and conversely, interventions that signal staffs’ trust of patients, such as engaging in boxercise with patients (McEwan, McClarty, and Porter 2016), or that de‐segregate staff and patient spaces, like leaving the ward office door open (McKeown et al. 2020), have been established to reduce violence.

### 4.4 Over‐Reliance on Techniques

De‐escalation is often used as the first line of defence against violence on psychiatric wards and involves staff utilising a set of techniques at the point of conflict such as verbal and non‐verbal skills, maintaining personal control, understanding when to intervene (Bowers 2013; Lavelle et al. 2016). Research in this area tends to focus on de‐escalation training delivered to individual staff members, which have limited effectiveness (Price et al. 2015).

Consequently, when vulnerability is discussed in this context, it is often assimilated to a skill or technique that can be utilised in conflict situations. This approach does not provide an account of vulnerability in and of itself, but of the potential benefit of expressions of vulnerability as a way of de‐escalating tense situations by signalling hurt, anxiety or helplessness (Bachmann, Michaelsen, and Vatne 2016; Sanford and Rowatt 2004). Not only does approaching vulnerability in this way miss its primary value (to enable the co‐construction of mutual and close relationships) but patients tend to respond negatively to staff expressing emotion within conflict situations if this expression is not consonant with their existing relationship (Goodman et al. 2020). In general, techniques which staff learn and utilise are often viewed as inauthentic by patients because they do not entail a genuine responsiveness or openness to the situation, just a pre‐existing set of scripted responses.

Understood as a level of affective openness as well as an openness to change, vulnerability could be described as the anti‐technique; instead of offering a way in which staff can successfully exercise control over their environment, it involves a relinquishing of control and an opening up of ways to be changed by that environment. Importantly, this clearly distinguishes the practice of vulnerability from a model which involves staff utilising de‐escalation techniques as a way of reducing conflict and violence on psychiatric wards. Although de‐escalation techniques are preferable to restraint as a way of managing conflict, because they reduce immediate risk to patients of both physical harm and traumatisation (Haefner, Dunn, and McFarland 2021) and have gained widespread usage because technique‐based training is easier and cheaper to deliver (Lyon et al. 2011), there are still problems in basing patient/staff relationships solely on the considered application of techniques. This does not mean that techniques have no role in building authentic relationships—for example learning strategies for communicating needs to a partner could improve and deepen the connection you have with them—however, within inpatient psychiatric settings they are often used instead of a relationship, as opposed to a means to developing one. Overreliance on techniques could be exacerbated by relying on bank or agency nursing staff, who might not know the patients they are working with.

Several defining features of techniques make them potentially alienating, harmful or inappropriate when used in this way. Firstly, the scripted nature of techniques is often seen by patients as robotic, inauthentic and uncompassionate, since it does not originate in a genuine response to patients (Augustus, Patrick, and Gardner 2022). Secondly, they have high fungibility: staff use them with anyone regardless of the specific relationship a staff member has with that patient, and different staff use the same techniques, making the relationship (or even the specific patient) irrelevant to their application. Finally, the idea of a technique reinforces a dynamic where staff members are acting subjects and the patients are objects who are unilaterally acted on. From the perspective of relational ethics, heavy context‐less use of techniques is relationship‐withering, compromising a therapeutic relationship or undermining the possibility of its development.

These features of techniques set them in contrast to the practice of vulnerability. Vulnerability involves an opening up, unmasking and sharing of someone's authentic self (to greater or lesser extent depending on context), arises spontaneously in response to specific features of the situation the person is in, who they are, and the individual they are relating to, and finally is fundamentally reciprocal and collaborative in that expressions of vulnerability from staff members can be met with, and deepened as a result of, an expression of vulnerability from patients.

### 4.5 Staff Infallibility

Given a broader view of vulnerability as openness to transformation, staff infallibility also presents a roadblock to practicing vulnerability and developing therapeutic relationships rooted in more mutual forms of vulnerability. In contrast, a desirable form of fallibility consists of being open to the possibility of changing your perspective in response to an encounter with another's as well as to changing your sense of self. Such fallibility or humility may be challenging for staff, or rejected by them, for various reasons: because their social position contrasts them as the competent professionals with patients regarded as incompetent, vulnerable dependents; because their sense of self is thus established in opposition to the perceived identities of patients, in terms of competence and professionalism rather than vulnerability and deficiency; because professionalism and expertise are often associated with not changing one's mind, not doubting, not hesitating rather than second‐guessing, revision, and transformation.

Empirical research often highlights the importance of flexibility to de‐escalation, a skill that can involve anything from communicating with patients in a way they find helpful (even if this is perceived as unprofessional), to subverting ward rules and practices, avoiding unnecessary limit‐setting and going against the decisions of colleagues to respond to patient needs (Bowers 2009; Bowers et al. 2009; Duxbury et al. 2019). Although flexibility tends to be framed as a skill or technique to be applied in conflict situations, it fits poorly within this framework. Either the skill consists of staff abilities to self‐present as flexible or reasonable, or it involves a deeper change of position in response to discussions and encounters with a patient's viewpoint, a relational practice that can be much better understood utilising the conception of vulnerability we argue for in this paper. Under this viewpoint, staff admitting fallibility would constitute a form of epistemic vulnerability—an acknowledgement both of your own limited capacity as a knower and the potential for being wrong, but also of epistemic fallibility on the part of the institution; just because something is an established rule or practice, does not mean it is the best way of doing things, or even a good way of doing things. Merely simulating this process, appearing to patients as reasonable and understanding without being prepared to change your perspective as a result of engaging with them, is problematic on two levels. At best, it removes the possibility for growth, change and improvement (on an institutional and personal level) as a result of staff encounters with patients, marginalising patient perspectives, limiting their power to effect positive change, and contributing to the epistemic injustice suffered by psychiatric patients (Crichton, Carel, and Kidd 2017). At worst, repeatedly appearing to change your perspective to take on that of another, without any evident sustained change occurring as a result, seems likely to decrease patients’ trust in staff even further. Epistemic vulnerability not only involves changing and developing as a result of relational encounters, but also expressing that change by apologising and sincerely admitting wrong‐doing; something particularly emphasized as important following use of restraint and seclusion (Berring, Pedersen, and Buus 2016).

### 4.6 Ethical Blunting

Failure to engage emotionally whilst working on psychiatric wards has an additional consequence of preventing staff from developing an ethical awareness of the harms of restrictive practices. A lack of awareness could result in a decreased motivation not to engage in these practices to minimise the harms to patients (and staff) brought about by seclusion and restraint. Restraint is a practice that often causes significant trauma to patients, both in and of‐itself and by re‐traumatising them through replicating previous abuse experiences (Frueh et al. 2005; Robins et al. 2005). There is a substantial movement within psychiatric nursing to promote, and give an account of, the possibility of conscientious objection in response to requests to restrain patients (Herrera 2020; Nussbaum and Wynia 2020; Petrini 2013), based on the harm caused to patients by this practice and the conflict between that harm and the values and principles of nursing staff.

However, full acknowledgement of the harms caused by this practice can be blocked by nurses’ attempts to avoid the vulnerability that accompanies emotional openness or empathy, remaining closed off from their own and others emotions (Herrera 2020). This prevents staff's own experiences of suffering and vulnerability from shaping their ethical practice (Thorup et al. 2012), potentially resulting in increased justifications of seclusion and restraint by nursing staff (Palazzolo 2004; Petti et al. 2001).

## 5 Being Open in Closed Places

Staff's avoidance of vulnerability and their pursuit of invulnerability stems in part from the perceived requirement to preserve the power staff as a group hold over psychiatric inpatients. Removing the need for staff's pursuit of invulnerability requires changes that increase the power psychiatric patients have over their care (encompassing legal frameworks, organisational structures, and patient capacity to organise against oppression). However, this avoidance of vulnerability is not only caused by a power imbalance between staff and patients, but also contributes to it, and as such it is important to understand how changes could be made directly in how staff relate to their own vulnerability.

It is our approach that seeing vulnerability only as susceptibility to harm misses the fundamental value of this concept. This is not to deny that vulnerability can open individuals up to the increased possibility that others will harm them. Despite the long‐term costs, emotional blunting is an adaptive response to trauma (Meyer et al. 2016), reducing the possibility of being further emotionally harmed. Harms to staff aside, vulnerability involves exposure and can prompt fear, defensiveness, and the use of violence to counteract these ensuing negative emotions (Vandello et al. 2008). Staff feeling over‐exposed on wards has the potential to increase their use of restraint and seclusion to make themselves feel safer.

Although practices such as emotional suppression (adopted in the pursuit of invulnerability) are most associated with burnout for mental health staff (Edward, Hercelinskyj, and Giandinoto 2017) this does not mean that emotional vulnerability is not emotionally exhausting, or even harmful to staff. Even if a context supports practices associated with vulnerability, the extra emotional labour required to genuinely connect to and be vulnerable with both patients and themselves could be seen as an additional burden to staff, on top of other stresses and burdens incurred in their workplace (Hochschild 2012). There is a difficulty at the heart of requiring psychiatric staff to be vulnerable with patients as part of their job; staff/patient relationships are not voluntary for patients (particularly those detained in hospital), but they are not voluntary for staff either, who exist under conditions of economic coercion. Particularly for staff with lived experience of mental illness, it could be that relatively small amounts of emotional openness in the workplace could be too draining or burdensome. As we have argued above, coerced openness is damaging in a variety of ways, and it is not our view that staff should be required as a condition of their employment to be vulnerable in the workplace. Even staff voluntarily grappling with their own vulnerability might be harmed as a result (Edward, Hercelinskyj, and Giandinoto 2017). There need to be ways of articulating the limits of emotional vulnerability; these will be different not just for each member of staff, but within each relationship they have with both patients and other staff members.

In fact, there are numerous issues around vulnerability that inpatient psychiatric staff need to navigate, including developing an awareness of when vulnerability might be therapeutic to patients and when it might be inappropriate or harmful to either patients or staff. This awareness is not something that rules could be developed for in advance of the particularities of the context, staff member, and the relationships they have with people, but to a significant extent can only be learned through individual experience, and reflection on that experience. Senior and more experienced staff members have a significant part to play in role‐modelling vulnerability and teaching staff how to navigate their own vulnerability in a non‐harmful way (Keeling and Templeman 2013). However, it is also important for nursing staff to have spaces in which vulnerability can be cultivated and navigated. Schwartz rounds were developed initially to provide a unique space for medical students to 'share emotions and express vulnerability' (Barker, Cornwell, and Gishen 2016), however, they are increasingly used across disciplines in mental health settings, where there have been positive impacts of staff's feelings of connectedness and emotional wellbeing (Allen et al. 2020; Farr and Barker 2017). Such interventions appear promising as a permanent and frequent forum for staff to cultivate vulnerability with each other as potential precursors to its cultivation in relationships with patients. In addition, there is evidence to suggest that the inclusion of peer or lived‐experience roles on psychiatric wards has an impact of reducing stigma within the staff team, making other staff members more likely to be open about their experiences, especially with each other (Harris, Leskela, and Hoffman‐Konn 2016; Harris et al. 2019). These kinds of interventions do not just have value in enabling staff to cultivate vulnerability in a safe way without risking harm to themselves or patients, but they could also signal organisational attitudes to vulnerability, and go some way to reducing some of the stigmatisation of any vulnerability that occurs. Nursing staff's capacity to be vulnerable in places as violent and traumatic as psychiatric wards has obvious value. However, vulnerability as an emotional practice requires substantial structural, organisational, and systemic changes to flourish. Although cultivating vulnerability might not be possible, or even conceivable in the current climate of psychiatric wards, jettisoning habits of invulnerability (emotional suppression, separation from patients, aspiring to infallibility and reliance on techniques) is a move towards a culture of vulnerability and could provide a basis for future development even if it is not a cultivation of vulnerability as such. An 'ethics of vulnerability does not demand that all embrace vulnerability unqualifiedly and in all circumstances, but rather that habitual repudiation of vulnerability be renounced' since '[v]ulnerability is not an absolute value to be avowed, but a fundamental condition with which to reckon' (Gilson 2013, p. 145). There need to be places on psychiatric wards open enough to allow this reckoning to occur.

## Author Contributions

CP and IJ were responsible for the initial conceptualisation of the article and writing the first draft, EG provided supervision and oversight. All authors contributed to critically reviewing, editing, revising and writing latter drafts of the article. The authors did not receive support from any organisation for the submitted work.

## Conflicts of Interest

The authors declare no conflicts of interest.

## Data Availability

Data sharing is not applicable to this article as no new data were created or analysed in this study.
